# Professional quality of life of child welfare workers and psychotherapists working with traumatized young unaccompanied refugees in Germany: a cross-sectional study

**DOI:** 10.1186/s13034-025-00942-0

**Published:** 2025-07-15

**Authors:** Pia Maria Schwegler, Barbara Kasparik, Jonathan Thielemann, Rebekka Eilers, Elisa Pfeiffer, Cedric Sachser, Rita Rosner

**Affiliations:** 1 Department of Clinical and Biological Psychology, Catholic University Eichstätt-Ingolstadt, Eichstaett-Ingolstadt, 85072 Germany; 2 Department of Clinical Psychology and Child and Adolescent Psychotherapy, Catholic University Eichstätt-Ingolstadt, Eichstaett- Ingolstadt, Germany; 3 Department of Child and Adolescent Psychiatry/Psychotherapy, Ulm University, Ulm, 89070 Germany; 4 Department of Clinical Child and Adolescent Psychology, Institute of Psychology, University of Bamberg, Bamberg, Germany

**Keywords:** Child welfare workers, Psychotherapists, Unaccompanied young refugees, Compassion satisfaction, Burnout, Secondary traumatic stress, ProQOL

## Abstract

**Background:**

Burnout symptoms and secondary traumatic stress are especially high among Child Welfare Workers (CWWs) and psychotherapists and might have increased since the recent increase in refugee numbers. Little is known about the wellbeing of CWW and psychotherapists working with unaccompanied young refugees (UYR), especially in German child and youth welfare facilities where they work closely together. This study aims to assess levels of compassion satisfaction (CS), burnout (BO), and secondary traumatic stress (STS) in German CWWs and psychotherapists, examining connections to demographic variables and group differences.

**Methods:**

*N* = 198 CWW and *N* = 97 psychotherapists were assessed via the Professional Quality of Life Scale (ProQOL). Descriptive statistics, t-test for independent samples, correlations between the ProQOL scales and hierarchical regression analyses with demographic variables as predictors were computed.

**Results:**

Both samples showed average to high levels of CS, and low to average levels of BO and STS. CWWs scored significantly higher than psychotherapists on BO and STS. In CWW, demographic variables were not associated with CS, BO or STS. Among psychotherapists, previous experiences in working with UYR (β = 0.38; *p* <.001) were positively associated with CS. Factors such as weekly therapy sessions (β = − 0.35, *p* =.001) and the psychotherapist’s migration background (β = − 0.20, *p* =.048) were negatively associated with STS. The number of PTSD cases treated (β = 0.27, *p* =.018) and additional training (β = 0.31, *p* =.006) were positively associated with STS.

**Conclusion:**

Among psychotherapists, prior experience in working with UYRs may contribute to elevated levels of CS, whereas a higher number of previously treated PTSD cases appears to be associated with increased STS. Further research on the influence of sociodemographic variables is needed for CWWs to identify protective and risk factors. Supporting and training CWWs and psychotherapists is crucial for quality treatment of traumatized UYRs.

**Supplementary Information:**

The online version contains supplementary material available at 10.1186/s13034-025-00942-0.

## Introduction

According to the United Nations High Commissioner for Refugees (UNHCR) the number of refugees worldwide has increased over the years (currently 36.4 million) [1]. This includes unaccompanied refugee minors, who are often exposed to traumatic events during their flight [2]. In Germany, child welfare workers (CWW) and psychotherapists work closely together to support UYR.

In general, CWW and psychotherapists belong to occupational groups described as likely to experience psychological distress and emotional burden [3–6]. These stressful experiences may result in feelings of overwhelming responsibility and strained personal relationships [7]. In addition, the workload and high levels of organizational demands have further increased in the last years, and cases are often characterized by complexity and severity, resulting in a high emotional burden for CWW and psychotherapists [8]. Persistent psychological distress can lead to mental and physical exhaustion [9], exit-seeking behaviors and work withdrawal [10]. Moreover, the CWW’s daily contact with physically, sexually, and emotionally abused children puts them especially at risk to develop burnout (BO) symptoms, secondary traumatic stress (STS) or a lack of compassion satisfaction (CS) [8, 11–13].

For both, CWW and psychotherapists, a particular challenging field is the work with unaccompanied young refugees (UYR). In Germany, the number of UYR has increased [14] and studies have indicated a higher prevalence of mental disorders among UYR [15–19]. Thus, there is a great need for mental health interventions tailored to the needs of UYR [20]. In the work with refugees, the likelihood of secondary exposure to traumatic events is high for therapists and social workers. Research has shown that BO and STS symptoms are possible consequences of working with a highly traumatized population [21–24]. In their review, Roberts and colleagues [23] reported that individuals working professionally and voluntarily with forcibly displaced people had pooled prevalence rates of 29.7% for high-level BO and 45.7% for moderate to severe-level STS, respectively. In the work with refugees, specific demands include a heightened emotional impact of work, cultural or language barriers and additional administrative obstacles regarding their residence status and other refugee-specific topics [25]. Lastly, working with refugee children seems to be associated with higher STS scores compared to working with refugee adults [21].

For STS, studies with social workers have reported various risk factors, such as female gender, younger age, or years of work experience [8, 12, 26]. Moreover, with increasing years of experience, BO and STS increase while CS seems to decrease. However, an earlier study by Avieli and colleagues [27] showed in a sample of social workers and therapists that with the increase in years of experience, STS and BO levels decreased significantly, whereas CS levels increased. Thus, in terms of experience, it is unclear whether it should be considered a risk or protective factor and how this may differ depending on the professional role.

Regarding therapists, various risk factors are discussed [28, 29]. Yang and Hayes [30] reported risk factors for BO such as a lack of perceived job control, a demanding caseload, and psychotherapists’ mental health such as trauma history. It has also been found that personal trauma experience is a risk factor for compassion fatigue [31]. Moreover, a personal history of trauma and flight experience favors higher levels of STS [21]. Furthermore, Simionato and Simpson [29] found a significant correlation between younger age, having less work experience, and being overinvolved in client problems with moderate to high levels of BO. Regarding STS, some studies have shown that a greater number of patients with PTSD is associated with increased STS [32, 33], while CS appears to increase with work experience [27, 34].

Among professionals who provide therapeutic support to refugees, rapport, and supervision and particularly the alliance with the supervisor was related to well-being, such as professional growth, balance, and employing boundaries [35]. Furthermore, Denkinger and colleagues [21] found that a secure attachment style can function as a protective factor preventing STS in a sample of psychologists, psychotherapists, interpreters, social workers, volunteers, and others who provided treatment and support to refugees from Iraq. Among therapists who provide therapeutic services for traumatized refugees, higher rates of self-efficacy and trauma-specific training led to lower levels of STS [36]. In a study by Plakas and colleagues [37] prior educational level could not be identified as an influencing factor on CS, BO or STS among social workers, coordinators, fieldworkers, or others working with refugees. It has not been investigated whether the therapeutic background (i.e. CBT, depth psychology etc.) has an influence on STS, BO, and CS scores.

Although there appears to be an increase in research that captures the impact of working with refugees in social workers and mental health professionals, there is a lack of knowledge about the wellbeing of CWW and licensed psychotherapists working with UYR. However, this is particularly important as UYR rely more heavily on social workers than accompanied young refugees or refugee adults. Social workers and therapists frequently adopt a more parental position. The aim of the present study is to identify levels of CS, BO, and STS in German CWW and licensed psychotherapists, examine differences between the two groups of professionals, as well as to investigate the influence of demographic and work-related variables on these dimensions. Demographic and work-related variables vary depending on the respective group. For more detail see Sect. Statistical analysis.

## Materials and methods

### Ethical considerations

Written informed consent was obtained from all individual participants included in the study. The studies involving human participants were reviewed and approved by ethics committees at The Ulm University (No. 243/19) and at The Catholic University of Eichstätt-Ingolstadt (No. 004–19). All data collection is carried out in accordance with the relevant guidelines and regulations following the Declaration of Helsinki.

### Study design and data collection

Participant data were collected between 2020 and 2022 within a greater trial [38]. The main study aims at comparing a stepped care approach to a treatment as usual for UYR. Therefore, psychotherapists were recruited to provide trauma-focused cognitive behavioral therapy [39] to the UYR. The social workers were recruited for external assessments and the implementation of a trauma-focused group intervention “Mein Weg” [40] in the Child and Youth Welfare Service (CYWS) facility. CWWs were recruited through the CYWS facilities where they were employed. Recruitment strategies for CYWS facilities included letters of invitation and telephone contact, digital information events and flyers. A website was created, and press interviews were conducted [38]. Psychotherapists were recruited via email through professional associations and personalized letters. The inclusion criteria for psychotherapist participants were: (1) licensed as either child and adolescent psychotherapists, or psychological psychotherapists, (2) agreement to treat up to three refugees with TF-CBT and (3) comply with all other study regulations. Additional information on recruitment strategies and inclusion criteria can be found in the study protocol [38]. Levels of CS, BO, and STS of CWW and psychotherapist participating in the trial were assessed at baseline. The data presented here corresponds to the baseline assessment before any intervention for the participating UYR had started. Thus, this cross-sectional survey was conducted at a time where some of the psychotherapists may have never worked with refugees but had declared their willingness to treat refugees within the trial. Demographic data were collected using two different standardized questionnaires. There were questions that were only suitable for one group or the other (e.g. for psychotherapists, “On average, how many therapy sessions do you have per week?”).

All participants completed the Professional Quality of Life Scale (ProQOL) [41]. The ProQOL measures the positive and negative effects of working with people who have experienced extremely stressful events. The self-report inventory contains 30 items on a five-point-Likert-Scale (1 = never, 2 = rare, 3 = sometimes, 4 = often, 5 = very often). It consists of three scales: Compassion Satisfaction (10 items, e.g., “I get satisfaction from being able to help people”), Burnout (10 items, e.g., “I feel trapped by my job as a helper”) and Secondary traumatic stress (10 items, e.g., “I feel depressed because of the traumatic experiences of the people I help”). In the present sample, chronbach’s alpha for the scales are satisfactory (CS: α = 0.88, BO: α = 0.75, STS: α = 0.81). The scales were calculated according to the manual with a higher value on a scale meaning a higher expression of the respective dimension (range 10–50). Recommended cut-off scores are low (0–22), moderate (23–41) and high (42–50) [41].

### Participants

The sample (*N* = 374) was composed of two different groups: (1) CWW (*n* = 266) who worked in 47 different CYWS facilities, and (2) licensed psychotherapists (*n* = 108) participating in the abovementioned trial and working together in the care for UYR. 43 cases of the CWW were excluded from analysis due to a high amount of missing data and 24 cases were excluded due to outliers. Regarding the psychotherapists, 3 cases were excluded due to a high amount of missing data and 8 cases were excluded due to outliers. Missing items refer to the fact that less than 50% or none of the ProQOL items were completed. Moreover, some of the socio-demographic data were completely missing. This left an overall sample of *n* = 295, CWW (*n* = 198, age *M* = 35.42, *SD* = 10.82, 72.7% female) and psychotherapists (*n* = 97, age *M* = 44.90, *SD* = 8.62, 81.4% female). For further information about the participants, see Tables 1 and 2.

**Table 1:** Sociodemographic characteristics of the CWW sample

Characteristics	CWW (*N* = 198)	
Age	*M* = 35.42 (*SD* = 10.82)	
Sex	144 female (72.7%)	
Graduation (school)/educational level	95 (48%) 72 (36,4%) 29 (14.6%) 2 (1%)	GCE A-levels advanced technical college certificate GCSE O-levels secondary school leaving certificate
Study or training	111 study (56.1%)	
Additional training	40.4%	
Work experience in general (years)	*M* = 8.95 (*SD* = 7.77)	
Work experience in current facility (years)	*M* = 4.01 (*SD* = 3.54)	
Work experience with UYR (months)	*M* = 41.87 (*SD* = 32.03)	

*Note. *GCE A-levels = General Certificate of Education Advanced Level, GCSE O-levels = General Certificate of Secondary Education Ordinary Level, UYR = unaccompanied young refugees

**Table 2:** Sociodemographic characteristics of the psychotherapist sample

Characteristics	Psychotherapists (*N* = 97)	
Age	*M* = 44.90 (*SD* = 8.62)	
Sex	79 female (81.4%)	
Migration background	15,5%	3.1% born in a different country 10.3% parents born in a different country 2.1% one parent has another citizenship)
Professional Background	37.1% pedagogy 28.9% psychology 21.6% social work 21.6% social pedagogy 9.3% teaching	
Treatment clientele/acquisition of the license to practice medicine	92.8% licensed psychotherapist for children and youth 5.2% licensed psychotherapist for adults 5.2% licensed psychotherapist for adults and children/youth Duration in years since the acquisition of the license to practice medicine: *M* = 7.87 (*SD* = 6.29)	
Additional training	70.1%	16.5% systemic therapy, 33% trauma therapy, 10.3% schema therapy, 32% EMDR, 7.2% hypnotherapy
Therapy sessions per week	*M* = 22.77 (*SD* = 7.72)	
Work experience with UYR	63.9%	
Number of PTSD patients that have already been treated	*M* = 13.68 (*SD* = 15.81)	
Therapeutic approach	Behavioral Therapy = 85.6%, Depth Psychology = 13.4%, others = 1%	
Patients to treat	Children and Youth = 92.8%, Adults = 5.2%, both = 5.2%	

### Statistical analyses

All analyses were performed using IBM SPSS Statistics (Version 28.0.0.0). Alpha level of statistical significance was set at *p* <.05 for all analyses. Outliers were identified using studentized excluded residuals, Cook’s distance, and leverage values [42, 43]. Means and standard deviations were calculated for all variables. Level of CS, BO and STS were also calculated and interpreted using cut-off scores [41]. Partial correlations between ProQOL scales were calculated. Due to the missing prerequisite of normal distribution in the STS score, the correlation with the other scales was performed using Kendall-Tau-b. Bias-corrected and accelerated bootstrap 95% CIs were used. A t-test for independent samples was performed to detect significant differences between the two groups (CWW and psychotherapists) regarding CS, BO, and STS. Additionally, the Mann-Whitney-U test was performed due to the lack of a normal distribution of the STS data from the CWW.

Hierarchical regression analyses were conducted for each of the three factors CS, BO and STS and groups (CWW and psychotherapists). Regression models were estimated for both groups based on previous research findings. The prerequisites for the hierarchical regression analyses were checked and implemented in advance. As presented in Table 3, sociodemographic and work-related variables were included block wise as independent variables in the hierarchical regression analyses.

**Table 3:** Block wise added variables of the hierarchical regression

Group	Model	Variables included
CWW	Model 1	Age
	Model 2	Gender
	Model 3	Years of work experience, experience with UYRs
	Model 4	Education level, training or academic study, further education
Psychotherapist	Model 1	Age, gender, migration background
	Model 2	Years since license acquisition, previous work with refugees, number of PTSD cases, sessions per week
	Model 3	Training in trauma therapy, schema therapy, hypnotherapy, systemic therapy
	Model 4	Professional background in psychology, social work, teaching, pedagogy, social pedagogy
	Model 5	Therapeutic approaches in depth psychology, behavioral therapy
	Model 6	Work with children and youth, work with adults

## Results

Descriptive data of the ProQOL scores as well as the correlations of the ProQOL scales for both groups can be found in Tables 4 and 5.

**Table 4:** Descriptive data of the ProQOL scores for CWW and psychotherapists

	CWW (*n* = 198)						Psychotherapists (*n* = 97)					
	*M*	*SD*	Range	Low	Moderate	High	*M*	*SD*	Range	Low	Moderate	High
CS	39.72	4.22	29–49	0%	64.6%	35.4%	40.06	4.10	29–49	0%	60.8%	39.2%
BO	21.94	4.18	13–34	59.1%	40.9%	0%	19.10	3.55	11–28	80.4%	19.6%	0%
STS	21.17	4.12	13–35	67.2%	32.8%	0%	18.12	3.43	12–26	89.7%	10.3%	0%

*Note.* CS = compassion satisfaction, BO = burnout, STS = secondary traumatic stress

**Table 5:** Correlations (r) of the ProQOL scores for CWW and psychotherapists

	CWW (*n* = 198)			Psychotherapists (*n* = 97)		
	CS	BO	STS	CS	BO	STS
CS	1	− 0.475** [−0.571, − 0.374]	− 0.110* [−0.220, − 0.010]	1	− 0.524** [−0.660, − 0.376]	− 0.200* [−0.388, 0.001]
BO	− 0.475**	1	0.363** [0.268, 0.448]	− 0.524**	1	0.514** [0.319, 0.692]
STS	− 0.110*^a^	0.363***^a^	1	− 0.200*	0.514**	1

*Note*. ** *p* <.001 und * *p* <.05. BCa bootstrap 95% CIs reported in brackets. CS = compassion satisfaction, BO = burnout, STS = secondary traumatic stress. ^a^ correlation was performed using Kendall-Tau-b

### Differences between CWW and psychotherapists

Regarding the BO scale, there was a significant difference between the BO scores of the psychotherapists (*M* = 19.10; *SD* = 3.55; *n* = 97) as compared to CWW (*M* = 21.94; *SD* = 4.18; *n* = 198), with mean difference of −2.84 (95%-CI[−3.81, −1.87]) lower for the psychotherapists, *t*(293) =. −5.75, *p* <.001. It did represent a medium-sized effect, *d* = −0.71. In addition, there was a significant difference between STS of psychotherapists (*M* = 18.12; *SD* = 3.43; *n* = 97) and the CWW (*M* = 21.17; *SD* = 4.12; *n* = 198), with mean difference of −3.05 (95%-CI [−4.00, −2.10]) lower for the psychotherapists, *t*(293) = −6.30, *p* <.001. It did represent a medium-sized effect, *d* = −0.78. The Mann-Whitney-U-test showed that the STS levels in psychotherapists (*Mdn* = 19.10) differ significantly from CWW, (*Mdn* = 21.17), *U* = 5712.00, *Z* = − 5.67, *p* <.001. There was no significant difference between CS scores of the psychotherapists (*M* = 40.06; *SD* = 4.10; *n* = 97) and the CWW (*M* = 39.72; *SD* = 4.22; *n* = 198), with mean difference of 0.34 (95%-CI [−0.68, 1.35]), *t*(293) = 0.656, *p* =.513, *d* = 0.08.

Additional analysis revealed a significant difference between CS scores of psychotherapists who have worked with refugees before (*M* = 41.24; *SD* = 3.87; *n* = 68) and those who have not (*M* = 37.92; *SD* = 4.21; *n* = 37), with mean difference of −3.32 (95%-CI [−4.93, −1.7]), *t*(103) = −4.07, *p* <.001, *d* = − 0.83. There were no significant differences regarding BO and STS. Comparing CS scores of psychotherapists with therapy experience working with refugees and CWW, CWW reported significant lower CS levels with mean difference of −1.42 (95%-CI [−2.62, − 0.23]), *t*(258) = 0.654, *p* =.02, *d* = − 0.34.

### Results of hierarchical regression analysis regarding demographic variables and ProQOL subscales

Hierarchical regression analyses for CWW can be found in Table S1 (additional file 1). All predictors were non-significant.

Detailed information on the models of the hierarchical regression for psychotherapists can be found in the additional file 1 (Table S2). Regarding the association between demographic variables and ProQOL subscales among psychotherapists, model 3 was able to explain the greatest significant amount (27%) of variance in CS [*F*(13, 83) = 2.35, *p* =.01]. The model revealed previous work with UYR as a significant predictor for CS (β = 0.34; *p* =.002).

Furthermore, model 6 was able to explain the greatest significant amount (39.4%) of variance in STS [*F*(22, 74) = 2.18, *p* =.007]. The analysis revealed that a higher number of weekly therapy sessions was associated with lower levels of STS (β = − 0.35, *p* =.002). Treating more cases diagnosed with PTSD was associated with higher levels of STS (β = 0.26, *p* =.041). Participants who had undergone additional training with a focus on trauma therapy reported higher STS levels (β = 0.34, *p* =.009). Participants with a migration background reported less STS (β = − 0.20, *p* =.05). There was no significant predictor for BO (see additional file 1, Table S2).

## Discussion

The aim of the present study was to identify levels of CS, BO, and STS in German CWW who work with UYR and licensed psychotherapists and to determine whether there was a difference between these two groups. Furthermore, we investigated whether demographic variables were associated with different levels in the ProQOL dimensions. In our overall sample, the participants reported average levels of BO and STS. Previous studies reported more average- to high-level scores on BO and STS among caregivers, refugee relief workers, professionals, and volunteers [21–24], whereas in our samples, mainly below average to average and no above average scores on BO and STS were found. The CS scores ranged from average to above average. Given that participants were likely already highly interested in working with refugees, CS levels may be elevated. Consequently, the sample mean may exceed that of the general population and should be taken into account when interpreting the results. Furthermore, differences in BO and STS scores between this and other studies may be due to differences in the areas of work. For example, working in receptive and transit centers [24] or working exclusively with women and children who may have experienced sexualized violence [21]. The social workers in our study worked in residential youth groups and the psychotherapists treated UYR alongside people without experience of flight and trauma. Additionally, 36.1% of therapists had not worked with refugees by the time the data was collected. Based on this study, future research should distinguish between different professional groups and areas of work with refugees.

Although the difference was small, psychotherapists scored significantly lower on the BO and STS scales than CWW. Little is known about the differences between professional groups working with refugees or UYR. Živanović and Marković [24] could not find any difference between occupational groups providing different types of services to refugees, i.e. legal, psychological, medical, or translational services. However, it seems that occupational stress is higher in CWW than in other professions due to various factors like higher caseloads, time pressure, being short staffed, working overtime, administrative regulations and protecting child safety [3, 4]. In our sample, the reason for the differences could be the nature and intensity of the contact. While CWW accompany children and young people in their everyday lives for several hours a day, up to 5 days a week, psychotherapists only see their clients in one to two hour sessions on a weekly basis. Another possibility are differences in training. Therapists learn about the treatment of PTSD during their undergraduate and postgraduate training, whereas social workers receive less intensive training. However, being trained in therapies with a traumafocus was associated with higher STS in our sample. It should also be noted that our group of psychotherapists had not all worked with UYR or carried out trauma therapy at the time of the analysis. Conversely, comparing therapists who have worked with refugees to those who have not revealed no differences in BO or STS scores. The analysis revealed that therapists with experience treating refugees had higher CS scores than those without. This suggests a positive effect associated with this work. These therapists also scored significantly higher on CS than the CWW group, which may reflect professional differences or statistical effects due to the groups’ different sample sizes. If these are professional differences, adapting labor policies to address challenges such as overtime, staff shortages, and high case numbers [3, 4] may reduce BO and STS while improving CS among CWW. However, as this is a cross-sectional survey, the generalizability of the results is limited. A longitudinal study is necessary to observe changes in CS, BO, and STS over time.

Surprisingly, there were no significant predictors in the hierarchical regression for CWW in all models. This finding is contrary to earlier studies that found that female gender and younger age are risk factors for BO and STS [8, 12]. However, in terms of work experience, our results fit the literature as some studies suggest that it puts CWW at risk [8, 26], while others indicate it as a protective factor [27]. When considering a floor effect, given the somewhat lower scores on the ProQOL scales in our sample, it may be that the gender and age did not come into effect. It may also be necessary to consider variables other than those we included. One possible predictor could be personal trauma history, as other studies have shown that CWW’s own trauma history has an impact on CS and STS [21, 31]. Furthermore, we included the predictors work experience in years and in the current facility. However, it might be more important to consider working hours, shift times or overtime, as these may better represent the workload that could be experienced as a burden or cause emotional distress [6]. It is possible that CWW seek intervisonary support from their colleagues. Support from team members has been reported to be essential for well-being [35] and may be a protective factor not captured by the variables in our study.

Among psychotherapists, hierarchical regression results indicated that higher numbers of weekly therapy sessions were associated with lower levels of STS. In this regard, studies are inconsistent, as in some, a positive correlation between the proportion of time spent with traumatized clients and the development of STS was shown [21], while in others, the opposite [44] or no correlation [24] was found. It should be noted that in the present study, we only asked about the number of therapy sessions per week, not specific to PTSD.

The observed association between the number of PTSD cases treated and increased STS scores warrants further investigation. It seems that it is not necessarily the number of patients per week that increases the STS score, but rather the total number of PTSD cases that are treated. These findings are consistent with those of Hensel and colleagues [33], who indicated that the proportion of time spent working with traumatized patients may be more important than the actual number of patients. When selecting cases, psychotherapists should consider this connection and arrange for parallel supervision when treating a high volume of trauma patients. Furthermore, future research should more rigorously collect data on the proportion of traumatized clients treated, trauma characteristics and treatment approaches to further evaluate this aspect.

Unexpectedly, participants who had undergone additional training focused on trauma therapy reported higher levels of STS. Earlier research indicated that the specific trauma training or more education should lead to a lower burden [44]. It is possible that psychotherapists with trauma training also have greater interest in treating PTSD and therefore take on more PTSD cases on average. This explanation aligns with our result that a higher PTSD caseload was associated with more STS. However, the possibility of habituation is important to consider. Additionally, it is unclear how much supervision psychotherapist with advanced trauma training still received which may have an impact on their STS scores [35]. The literature agrees on the need for trauma training, education, and supervision in the field of working with refugees [21, 24, 31, 35]. Moreover, continued supervision remains essential even for experienced psychotherapists who have treated a higher number of trauma cases. It should not be assumed that increased clinical experience reduces the need for supervision.

Another result was that psychotherapists with a migrant background reported lower STS scores. One potential explanation is that language barriers or cultural differences are less pronounced, leading to greater understanding and openness. In addition, their own migration history could also lead to higher resilience and thus lower STS values. However, Denkinger and colleagues found that a person’s own history of flight is a risk factor for STS [21]. Therefore, flight experience and migration background must be considered separately. Furthermore, psychotherapists with a migration or refugee background should receive more support. Although their experiences could offer valuable insights to improve care for UYR, they remain underrepresented in research and should be more actively included.

Furthermore, the analysis showed that greater experience in working with refugees was associated with higher levels of CS. Additionally, there are no significant differences in BO and STS based on whether or not psychotherapists have previously treated refugees. This finding is not consistent with the findings of Mavratza and colleagues [31] who found that working with migrants had a negative impact on professional quality of life, which they attributed to a possible influence on the BO and compassion fatigue scales. Plakas [37] was also able to show that an increase in work experience in humanitarian aid was associated with an increase in BO and a decrease in CS. However, it should be noted that these studies did not exclusively survey psychotherapists but also social workers, nurses, nursing assistants, coordinators, field workers and more. Furthermore, it is possible that psychotherapists with a higher CS trait are more likely to work with refugees. However, these findings suggest that working with refugees can be professionally rewarding. Early-career and experienced psychotherapists alike should be encouraged to engage in this field and provided with the necessary support.

### Limitations

This study has some limitations. First, the sample size varied substantially depending on the group. Subsequently, some of the analyses contained only a few participants and missed statistical power. In addition, some of the psychotherapists (36.1%) had not yet worked with refugees at the time of the survey. To ensure generalizability, future research should survey only therapists who already have experience in working with UYR. Moreover, questions should have been asked about personal trauma histories and personal experiences of flight. Additionally, we did not assess supervisionary support in CWW or psychotherapists. This may be necessary for obtaining further information on work-related stress factors. Another aspect affecting the sample is the context of the project in which the data were collected. It aims to implement a low-threshold care service for UYR. The CYWS facilities as well as the psychotherapists who register with the project have a certain interest and motivation to work with this special cohort. Therefore, the possibility of a selection bias cannot be ruled out. Furthermore, the data is cross-sectional, whereas longitudinal data could have provided more accurate information on the development of professional quality of life in the work with UYR. Due to the explorative approach, the results can only be generalized to a limited extent. This should be considered when interpreting our results and reviewing them in future studies.

## Conclusion and implications

In sum, BO and STS were present in CWW and psychotherapists, but levels were lower than expected. Psychotherapists had lower STS and BO scores than CWW, suggesting greater exposure to stress for CWW. However, no significant predictors could be detected in the hierarchical regression of the CWW. Among psychotherapists, a greater number of PTSD cases was associated with higher STS scores. It has been shown that a greater number of weekly therapy sessions was linked to lower STS scores. Psychotherapists with trauma training reported higher levels of STS. Having a migration background was associated with lower STS values. Furthermore, psychotherapists with experience in working with refugees reported higher levels of CS than those without.

Given the aforementioned limitations, several implications for future research and professional development emerge. To ensure the quality of treatment and care, it is important to support and train CWW and psychotherapists working with refugees. By doing so, potential barriers or fears of treatment can be removed. This includes adequate trauma-specific training as well as training on the topic of flight in general. Training should be provided not only in clinical settings but also in pedagogical and educational areas to equip young adults in social professions with basic knowledge. Additionally, studies often reference the added stress of working with refugees. However, it is possible that working with UYR can also be fulfilling and not solely diminish professional quality of life. Future research should address the role of occupational groups, treatment approach, and migration background of the psychotherapist. Furthermore, identifying risk and protective factors among CWW is important. Predictors such as trauma history, workload, and the importance of supervision or intervision support should be considered. Research in the field of professionals working with UYR should increase as it could support professionals’ quality of life and ultimately benefit their patients.

## Electronic supplementary material

Supplementary Material 1: Table S1 and S2: Coefficient of the hierarchical regression analyses (CWW & psychotherapists). 

## Data Availability

Our data were collected by the research group and are not publicly available. The datasets used and/or analyzed during the current study are available from the corresponding author on reasonable request.

## Abbreviations

BO: Burnout
CS: Compassion Satisfaction
CWW: Child Welfare Workers
CYWS: Child and Youth Welfare Service
proQOL: Professional Quality of Life Scale
PTSD: Posttraumatic Stress Disorder
STS: Secondary Traumatic Stress
UYR: Unaccompanied Young Refugees
