# Supplementary material for: Professional quality of life of child welfare workers and psychotherapists working with traumatized young unaccompanied refugees in Germany: a cross-sectional study
Source: Child Adolesc Psychiatry Ment Health. 2025 Jul 15;19:77. doi: 10.1186/s13034-025-00942-0 (PMC12261614; doi:10.1186/s13034-025-00942-0)
Supplement: Supplementary file 1 — Supplementary Material 1: Table S1 and S2: Coefficient of the hierarchical regression analyses (CWW & psychotherapists). [file 13034_2025_942_MOESM1_ESM.docx]

Table S1

*Coefficient of the hierarchical regression analyses (CWW)*

|  | CS score | | | | | | BO score | | | | | | STS score | | | |  |  |
| --- | --- | --- | --- | --- | --- | --- | --- | --- | --- | --- | --- | --- | --- | --- | --- | --- | --- | --- |
|  | *b* | *SE B* | *β* | *p* | *R²* | *Adj. R²* | *b* | *SE B* | *β* | *p* | *R²* | *Adj. R²* | *b* | *SE B* | *β* | *p* | *R²* | *Adj. R²* |
| Step 1 |  |  |  |  | 0.004 | -0.001 |  |  |  |  | 0.000 | -0.005 |  |  |  |  | 0.013 | 0.008 |
| Constant | 40.635 | 1.028 |  | <.001 |  |  | 22.108 | 1.021 |  | <.001 |  |  | 19.621 | 1.000 |  | <.001 |  |  |
| Age | -0.026 | 0.028 | -0.066 | .354 |  |  | -0.005 | 0.028 | -0.012 | .863 |  |  | 0.044 | 0.027 | 0.115 | .107 |  |  |
| Step 2 |  |  |  |  | 0.006 | -0.004 |  |  |  |  | 0.014 | 0.004 |  |  |  |  | 0.015 | 0.005 |
| Constant | 40.331 | 1.184 |  | <.001 |  |  | 23.077 | 1.168 |  | <.001 |  |  | 19.307 | 1.152 |  | <.001 |  |  |
| Age | -0.024 | 0.028 | -0.063 | .383 |  |  | -0.009 | 0.028 | -0.023 | .743 |  |  | 0.045 | 0.027 | 0.119 | .098 |  |  |
| Gender | 0.352 | 0.677 | 0.037 | .603 |  |  | -1.122 | 0.668 | -0.120 | .095 |  |  | 0.363 | 0.659 | 0.039 | .582 |  |  |
| Step 3 |  |  |  |  | 0.01 | -0.016 |  |  |  |  | 0.025 | 0.000 |  |  |  |  | 0.037 | 0.012 |
| Constant | 40.647 | 1.448 |  | <.001 |  |  | 23.154 | 1.423 |  | <.001 |  |  | 18.508 | 1.395 |  | <.001 |  |  |
| Age | -.049 | 0.049 | -0.125 | .323 |  |  | -0.025 | 0.048 | -0.066 | .601 |  |  | 0.077 | 0.047 | 0.201 | .107 |  |  |
| Gender | 0.019 | 0.685 | 0.034 | .636 |  |  | -1.131 | 0.673 | -0.121 | .094 |  |  | 0.428 | 0.660 | 0.046 | .517 |  |  |
| Work experience (years) | 0.019 | 0.067 | 0.035 | .778 |  |  | -0.008 | 0.066 | -0.014 | .909 |  |  | -0.080 | 0.065 | -0.151 | .216 |  |  |
| Work experience (current facility) | 0.044 | 0.111 | 0.037 | .694 |  |  | 0.133 | 0.109 | 0.113 | .223 |  |  | 0.199 | 0.107 | 0.171 | .065 |  |  |
| Work experience (UYR) | 0.005 | 0.012 | 0.040 | .660 |  |  | 0.001 | 0.012 | 0.007 | .938 |  |  | -0.011 | 0.012 | -0.082 | .365 |  |  |
| Step 4 |  |  |  |  | 0.019 | -0.022 |  |  |  |  | 0.033 | -0.008 |  |  |  |  | 0.049 | 0.009 |
| Constant | 39.688 | 2.003 |  | <.001 |  |  | 24.556 | 1.970 |  | <.001 |  |  | 18.585 | 1.926 |  | <.001 |  |  |
| Age | -0.048 | 0.050 | -0.122 | .341 |  |  | -0.029 | 0.049 | -0.076 | .553 |  |  | 0.067 | 0.048 | 0.117 | .162 |  |  |
| Gender | 0.474 | 0.744 | 0.050 | .524 |  |  | -1.019 | 0.732 | -0.109 | .165 |  |  | 0.279 | 0.715 | 0.030 | .696 |  |  |
| Work experience (years) | 0.001 | 0.069 | 0.001 | .992 |  |  | -0.006 | 0.068 | -0.010 | .935 |  |  | -0.077 | 0.067 | -0.146 | .249 |  |  |
| Work experience (current facility) | 0.044 | 0.112 | 0.037 | .696 |  |  | 0.136 | 0.110 | 0.115 | .219 |  |  | 0.185 | 0.108 | 0.159 | .088 |  |  |
| Work experience (UYR) | 0.004 | 0.012 | 0.032 | .730 |  |  | 0.000 | 0.012 | 0.003 | .970 |  |  | -0.010 | 0.012 | -0.080 | .380 |  |  |
| education level | 0.470 | 0.516 | 0.084 | .364 |  |  | -0.345 | 0.508 | -0.062 | .498 |  |  | -0.177 | 0.497 | -0.032 | .723 |  |  |
| Training or study | 0.260 | 0.754 | 0.031 | .730 |  |  | -0.802 | 0.742 | -0.097 | .281 |  |  | 0.095 | 0.725 | 0.012 | .895 |  |  |
| Further education | 0.709 | 0.655 | 0.083 | .280 |  |  | 0.351 | 0.644 | 0.041 | .586 |  |  | 0.870 | 0.630 | 0.104 | .169 |  |  |
| *Note.* *b* = regression coefficient, *SE B* = standard error, *β* = standardized coefficient beta, *p* = significance, *R²* = correlation coefficient, *adj. R²* = adjusted correlation coefficient. CS = compassion satisfaction, BO = burnout, STS = secondary traumatic stress. | | | | | | | | | | | | | | | | | | |

Table S2
*Coefficient of the hierarchical regression analyses (psychotherapists)*

|  | CS score | | | | | | BO score | | | | | | STS score | | | | | | | | | | | | | | | | | | | | |  |  |
| --- | --- | --- | --- | --- | --- | --- | --- | --- | --- | --- | --- | --- | --- | --- | --- | --- | --- | --- | --- | --- | --- | --- | --- | --- | --- | --- | --- | --- | --- | --- | --- | --- | --- | --- | --- |
|  | *b* | *SE B* | *β* | *p* | *R²* | *Adj. R²* | *b* | *SE B* | *β* | *p* | *R²* | *Adj. R²* | | *b* | | | *SE B* | *β* | | | | | *p* | | | | *R²* | *Adj. R²* | | | | |  |  |  |
| Step 1 |  |  |  |  | .036 | .005 |  |  |  |  | .03 | -.001 | |  | |  | | | |  | |  | | | | .08 | | | | | .05 | | | | |
| Constant | 37.089 | 2.341 |  | <.001 |  |  | 21.485 | 2.031 |  | <.001 |  |  | | 14.372 | | 1.911 | | | |  | | <.001 | | |  | | | | |  | | | |  |  |
| Age | 0.033 | 0.049 | .070 | .496 |  |  | -0.027 | 0.042 | -.066 | .519 |  |  | | 0.061 | | 0.040 | | | | .152 | | .129 | | |  | | | | |  | | | |  |  |
| Gender | 1.789 | 1.080 | .170 | .101 |  |  | -1.388 | 0.937 | -.153 | .142 |  |  | | 1.568 | | 0.882 | | | | .179 | | .079 | | |  | | | | |  | | | |  |  |
| Migration background | 0.160 | 1.159 | .014 | .891 |  |  | -0.173 | 1.006 | -.018 | .864 |  |  | | -1.607 | | 0.947 | | | | -.170 | | .093 | | |  | | | | |  | | | |  |  |
| Step 2 |  |  |  |  | .177 | .112 |  |  |  |  | .105 | .035 | |  |  | | | |  | |  | | | .22 | | | | | .159 | | |  |  |  |  |
| Constant | 35.465 | 2.822 |  | <.001 |  |  | 25.127 | 2.545 |  | <.001 |  |  | | 18.723 | | 2.295 | | | |  | | <.001 | | |  | | | | |  | | | |  |  |
| Age | 0.014 | 0.063 | .030 | .821 |  |  | -0.066 | 0.057 | -.160 | .250 |  |  | | 0.036 | | 0.052 | | | | .090 | | .491 | | |  | | | | |  | | | |  |  |
| Gender | 2.105 | 1.052 | .200 | **.049** |  |  | -1.816 | 0.949 | -.200 | .059 |  |  | | 0.967 | | 0.856 | | | | .110 | | .261 | | |  | | | | |  | | | |  |  |
| Migration Background | 0.034 | 1.111 | .003 | .976 |  |  | 0.127 | 1.002 | .013 | .899 |  |  | | -1.175 | | 0.904 | | | | -.125 | | .197 | | |  | | | | |  | | | |  |  |
| Years since acquisition of license | 0.037 | 0.094 | .057 | .696 |  |  | 0.081 | 0.085 | .143 | .344 |  |  | | 0.051 | | 0.077 | | | | .093 | | .510 | | |  | | | | |  | | | |  |  |
| Previous work with refugees | 3.233 | 0.882 | .380 | **<.001** |  |  | -0.831 | 0.795 | -.113 | .298 |  |  | | -0.363 | | 0.717 | | | | -.051 | | .614 | | |  | | | | |  | | | |  |  |
| Number of PTSD cases | -.027 | 0.029 | -.103 | .366 |  |  | 0.025 | 0.026 | .112 | .345 |  |  | | 0.039 | | 0.024 | | | | .179 | | .108 | | |  | | | | |  | | | |  |  |
| Therapy sessions per week | 0.011 | 0.057 | .020 | .852 |  |  | -0.090 | 0.051 | -.195 | .082 |  |  | | -0.154 | | 0.046 | | | | -.346 | | **.001** | | |  | | | | |  | | | |  |  |
| Step 3 |  |  |  |  | .269 | .154 |  |  |  |  | .169 | .039 | |  | |  | | | |  | |  | | | .347 | | | | | .245 | | | |  |  |
| Constant | 36.503 | 2.836 |  | <.001 |  |  | 25.718 | 2.615 |  | <.001 |  |  | | 19.877 | | 2.240 | | | |  | | <.001 | | |  | | | | |  | | | |  |  |
| Age | 0.022 | 0.066 | .047 | .737 |  |  | -0.090 | 0.061 | -.219 | .142 |  |  | | 0.014 | | 0.052 | | | | .035 | | .791 | | |  | | | | |  | | | |  |  |
| Gender | 1.147 | 1.076 | .109 | .290 |  |  | -1.696 | 0.992 | -.187 | .091 |  |  | | 0.915 | | 0.850 | | | | .104 | | .285 | | |  | | | | |  | | | |  |  |
| Migration background | 0.553 | 1.128 | .049 | .625 |  |  | -0.258 | 1.040 | -.026 | .804 |  |  | | -1.742 | | 0.891 | | | | -.185 | | .054 | | |  | | | | |  | | | |  |  |
| Years since acquisition of license | 0.084 | 0.099 | .129 | .396 |  |  | 0.096 | 0.091 | .170 | .295 |  |  | | 0.040 | | 0.078 | | | | .073 | | .613 | | |  | | | | |  | | | |  |  |
| Previous work with refugees | 2.902 | 0.887 | .342 | **.002** |  |  | -0.774 | 0.817 | -.105 | .346 |  |  | | -0.357 | | 0.700 | | | | -.050 | | .612 | | |  | | | | |  | | | |  |  |
| Number of PTSD cases | -0.044 | 0.031 | -.170 | .156 |  |  | 0.042 | 0.028 | .189 | .139 |  |  | | 0.058 | | 0.024 | | | | .269 | | **.018** | | |  | | | | |  | | | |  |  |
| Therapy sessions per week | 0.011 | 0.056 | .020 | .848 |  |  | -0.078 | 0.052 | -.171 | .132 |  |  | | -0.144 | | 0.044 | | | | -.325 | | **.002** | | |  | | | | |  | | | |  |  |
| AT (trauma therapy) | 0.563 | 1.011 | .065 | .579 |  |  | 1.424 | 0.932 | .190 | .130 |  |  | | 2.269 | | 0.798 | | | | .313 | | **.006** | | |  | | | | |  | | | |  |  |
| AT (schema therapy) | -2.580 | 1.382 | -.192 | .066 |  |  | -0.775 | 1.275 | -.067 | .545 |  |  | | -0.267 | | 1.092 | | | | -.024 | | .807 | | |  | | | | |  | | | |  |  |
| AT (hypnotherapy) | -3.123 | 1.607 | -.198 | .055 |  |  | -0.424 | 1.482 | -.031 | .776 |  |  | | -0.901 | | 1.269 | | | | -.068 | | .480 | | |  | | | | |  | | | |  |  |
| AT (systemic therapy) | -1.019 | 1.206 | -.093 | .400 |  |  | 0.638 | 1.112 | .067 | .568 |  |  | | 1.725 | | 0.953 | | | | .188 | | .074 | | |  | | | | |  | | | |  |  |
| AT (EMDR) | 1.385 | 1.123 | .158 | .221 |  |  | -1.803 | 1.035 | -.238 | .085 |  |  | | -1.544 | | 0.887 | | | | -.211 | | .085 | | |  | | | | |  | | | |  |  |
| AT (yes / no) | -0.838 | 1.129 | -.094 | .460 |  |  | -0.138 | 1.041 | -.018 | .895 |  |  | | -1.270 | | 0.892 | | | | -.170 | | .158 | | |  | | | | |  | | | |  |  |
| Step 4 |  |  |  |  | .282 | .116 |  |  |  |  | .175 | -.015 | |  | |  | | | |  | |  | | | .365 | | | | | .218 | | | |  |  |
| Constant | 35.551 | 3.394 |  | <.001 |  |  | 25.960 | 3.147 |  | <.001 |  |  | | 19.994 | | 2.669 | | | |  | | <.001 | | |  | | | | |  | | | |  |  |
| Age | 0.009 | 0.070 | .019 | .900 |  |  | -0.093 | 0.065 | -.225 | .157 |  |  | | 0.001 | | 0.055 | | | | .002 | | .989 | | |  | | | | |  | | | |  |  |
| Gender | 1.677 | 1.212 | .160 | .170 |  |  | -1.610 | 1.123 | -.177 | .156 |  |  | | 1.018 | | 0.953 | | | | .116 | | .289 | | |  | | | | |  | | | |  |  |
| Migration background | 0.479 | 1.157 | .042 | .680 |  |  | -0.306 | 1.073 | -.031 | .776 |  |  | | -1.749 | | 0.910 | | | | -.185 | | .058 | | |  | | | | |  | | | |  |  |
| Years since acquisition of license | 0.097 | 0.106 | .149 | .364 |  |  | 0.088 | 0.099 | .156 | .376 |  |  | | 0.073 | | 0.084 | | | | .134 | | .386 | | |  | | | | |  | | | |  |  |
| Previous work with refugees | 3.048 | 0.961 | .359 | **.002** |  |  | -0.840 | 0.891 | -.114 | .349 |  |  | | -0.442 | | 0.756 | | | | -.062 | | .560 | | |  | | | | |  | | | |  |  |
| Number of PTSD cases | -0.043 | 0.032 | -.164 | .182 |  |  | 0.042 | 0.029 | .189 | .152 |  |  | | 0.059 | | 0.025 | | | | .271 | | **.020** | | |  | | | | |  | | | |  |  |
| Therapy sessions per week | 0.005 | 0.059 | .010 | .930 |  |  | -0.085 | 0.054 | -.186 | .120 |  |  | | -0.148 | | 0.046 | | | | -.334 | | **.002** | | |  | | | | |  | | | |  |  |
| AT (trauma therapy) | 0.484 | 1.047 | .056 | .645 |  |  | 1.464 | 0.971 | .195 | .136 |  |  | | 2.213 | | 0.823 | | | | .305 | | **.009** | | |  | | | | |  | | | |  |  |
| AT (Schema therapy) | -2.925 | 1.450 | -.218 | **.047** |  |  | -0.918 | 1.345 | -.079 | .497 |  |  | | -0.338 | | 1.140 | | | | -.030 | | .767 | | |  | | | | |  | | | |  |  |
| AT (hypnotherapy) | -3.011 | 1.687 | -.191 | .078 |  |  | -0.300 | 1.564 | -.022 | .849 |  |  | | -0.908 | | 1.327 | | | | -.069 | | .496 | | |  | | | | |  | | | |  |  |
| AT (systemic therapy) | -1.275 | 1.280 | -.116 | .322 |  |  | 0.525 | 1.187 | .055 | .660 |  |  | | 1.444 | | 1.006 | | | | .157 | | .155 | | |  | | | | |  | | | |  |  |
| AT (EMDR) | 1.437 | 1.207 | .164 | .237 |  |  | -1.737 | 1.119 | -.229 | .125 |  |  | | -1.359 | | 0.949 | | | | -.186 | | .156 | | |  | | | | |  | | | |  |  |
| AT (yes / no) | -0.800 | 1.159 | -.090 | .492 |  |  | -0.084 | 1.074 | -.011 | .938 |  |  | | -1.317 | | 0.911 | | | | -.177 | | .152 | | |  | | | | |  | | | |  |  |
| PB (psychology) | 1.267 | 1.357 | .141 | .353 |  |  | 0.212 | 1.259 | .027 | .867 |  |  | | -0.405 | | 1.067 | | | | -.054 | | .706 | | |  | | | | |  | | | |  |  |
| PB (Social work) | 0.481 | 1.309 | .049 | .714 |  |  | -0.325 | 1.213 | -.038 | .790 |  |  | | 0.332 | | 1.029 | | | | .040 | | .748 | | |  | | | | |  | | | |  |  |
| PB (Teaching) | 0.786 | 1.759 | .056 | .656 |  |  | -0.726 | 1.631 | -.060 | .657 |  |  | | 0.465 | | 1.383 | | | | .040 | | .738 | | |  | | | | |  | | | |  |  |
| PB (Pedagogy) | 0.651 | 1.307 | .077 | .620 |  |  | 0.081 | 1.211 | .011 | .947 |  |  | | 0.322 | | 1.027 | | | | .046 | | .755 | | |  | | | | |  | | | |  |  |
| PB (Social pedagogy) | 1.448 | 1.370 | .146 | .294 |  |  | 0.399 | 1.270 | .047 | .754 |  |  | | 0.859 | | 1.077 | | | | .104 | | .427 | | |  | | | | |  | | | |  |  |
| Step 5 |  |  |  |  | .284 | .096 |  |  |  |  | .180 | -.035 | |  | |  | | | |  | |  | | | .383 | | | | | .22 | | | |  |  |
| Constant | 34.535 | 5.462 |  | <.001 |  |  | 28.252 | 5.055 |  | <.001 |  |  | | 18.444 | | 4.239 | | | |  | | <.001 | | |  | | | | |  | | | |  |  |
| Age | 0.016 | 0.076 | .034 | .834 |  |  | -0.108 | 0.070 | -.263 | .128 |  |  | | 0.012 | | 0.059 | | | | .031 | | .835 | | |  | | | | |  | | | |  |  |
| Gender | 1.771 | 1.250 | .169 | .161 |  |  | -1.766 | 1.157 | -.194 | .131 |  |  | | 1.203 | | 0.970 | | | | .137 | | .219 | | |  | | | | |  | | | |  |  |
| Migration background | 0.453 | 1.196 | .040 | .706 |  |  | -0.347 | 1.107 | -.036 | .755 |  |  | | -1.865 | | 0.929 | | | | -.198 | | **.048** | | |  | | | | |  | | | |  |  |
| Years since acquisition of license | 0.093 | 0.112 | .143 | .408 |  |  | 0.101 | 0.103 | .179 | .332 |  |  | | 0.070 | | 0.087 | | | | .128 | | .422 | | |  | | | | |  | | | |  |  |
| Previous work with refugees | 3.054 | 0.973 | .359 | **.002** |  |  | -0.836 | 0.901 | -.114 | .356 |  |  | | -0.417 | | 0.755 | | | | -.059 | | .583 | | |  | | | | |  | | | |  |  |
| Number of PTSD cases | -.041 | 0.033 | -.157 | .228 |  |  | 0.037 | 0.031 | .167 | .230 |  |  | | 0.061 | | 0.026 | | | | .281 | | **.021** | | |  | | | | |  | | | |  |  |
| Therapy sessions per week | 0.003 | 0.061 | .007 | .954 |  |  | -0.087 | 0.056 | -.190 | .125 |  |  | | -0.155 | | 0.047 | | | | -.350 | | **.002** | | |  | | | | |  | | | |  |  |
| AT (trauma therapy) | 0.514 | 1.153 | .059 | .657 |  |  | 1.560 | 1.067 | .208 | .148 |  |  | | 2.387 | | 0.895 | | | | .329 | | **.009** | | |  | | | | |  | | | |  |  |
| AT (schema therapy) | -3.058 | 1.531 | -.228 | **.049** |  |  | -0.659 | 1.417 | -.057 | .644 |  |  | | -0.576 | | 1.189 | | | | -.051 | | .630 | | |  | | | | |  | | | |  |  |
| AT (hypnotherapy) | -2.985 | 1.709 | -.189 | .085 |  |  | -0.311 | 1.582 | -.023 | .844 |  |  | | -0.831 | | 1.326 | | | | -.063 | | .533 | | |  | | | | |  | | | |  |  |
| AT (systemic therapy) | -1.280 | 1.296 | -.116 | .326 |  |  | 0.548 | 1.199 | .058 | .649 |  |  | | 1.445 | | 1.006 | | | | .157 | | .155 | | |  | | | | |  | | | |  |  |
| AT (EMDR) | 1.433 | 1.222 | .164 | .244 |  |  | -1.720 | 1.131 | -.227 | .132 |  |  | | -1.360 | | 0.948 | | | | -.186 | | .156 | | |  | | | | |  | | | |  |  |
| AT (yes / no) | -.812 | 1.186 | -.091 | .496 |  |  | -0.122 | 1.097 | -.016 | .912 |  |  | | -1.384 | | 0.920 | | | | -.186 | | .137 | | |  | | | | |  | | | |  |  |
| PB (psychology) | 1.323 | 1.380 | .147 | .341 |  |  | 0.168 | 1.278 | .022 | .896 |  |  | | -0.255 | | 1.071 | | | | -.034 | | .813 | | |  | | | | |  | | | |  |  |
| PB (social work) | 0.527 | 1.329 | .053 | .693 |  |  | -0.359 | 1.230 | -.042 | .771 |  |  | | 0.455 | | 1.032 | | | | .055 | | .660 | | |  | | | | |  | | | |  |  |
| PB (teaching) | 0.752 | 1.781 | .053 | .674 |  |  | -0.679 | 1.648 | -.056 | .682 |  |  | | 0.389 | | 1.382 | | | | .033 | | .779 | | |  | | | | |  | | | |  |  |
| PB (pedagogy) | 0.728 | 1.339 | .086 | .588 |  |  | 0.031 | 1.239 | .004 | .980 |  |  | | 0.537 | | 1.039 | | | | .076 | | .607 | | |  | | | | |  | | | |  |  |
| PB (social pedagogy) | 1.588 | 1.438 | .160 | .273 |  |  | 0.303 | 1.331 | .035 | .820 |  |  | | 1.247 | | 1.116 | | | | .151 | | .267 | | |  | | | | |  | | | |  |  |
| Therapeutic approach (depth psychology) | 0.092 | 2.977 | .008 | .975 |  |  | -0.979 | 2.756 | -.094 | .723 |  |  | | -0.469 | | 2.311 | | | | -.047 | | .840 | | |  | | | | |  | | | |  |  |
| Therapeutic approach (Behavioral therapy) | 0.680 | 2.864 | .059 | .813 |  |  | -1.541 | 2.651 | -.153 | .563 |  |  | | 1.032 | | 2.223 | | | | .106 | | .644 | | |  | | | | |  | | | |  |  |
| Step 6 |  |  |  |  | .292 | .081 |  |  |  |  | .19 | -.051 | |  | |  | | | |  | |  | | | .394 | | | | | .213 | | | |  |  |
| Constant | 35.277 | 5.983 |  | <.001 |  |  | 29.063 | 5.534 |  | <.001 |  |  | | 20.267 | | 4.627 | | | |  | | <.001 | | |  | | | | |  | | | |  |  |
| Age | 0.011 | 0.077 | .024 | .882 |  |  | -0.108 | 0.071 | -.262 | .133 |  |  | | 0.009 | | 0.059 | | | | .021 | | .886 | | |  | | | | |  | | | |  |  |
| Gender | 1.704 | 1.264 | .162 | .182 |  |  | -1.752 | 1.169 | -.193 | .138 |  |  | | 1.165 | | 0.977 | | | | .133 | | .237 | | |  | | | | |  | | | |  |  |
| Migration background | 0.508 | 1.208 | .045 | .676 |  |  | -0.375 | 1.117 | -.038 | .738 |  |  | | -1.858 | | 0.934 | | | | -.197 | | **.050** | | |  | | | | |  | | | |  |  |
| Years since acquisition of license | 0.109 | 0.114 | .167 | .344 |  |  | 0.085 | 0.106 | .151 | .424 |  |  | | 0.060 | | 0.088 | | | | .111 | | .497 | | |  | | | | |  | | | |  |  |
| Previous work with refugees | 2.808 | 1.020 | .330 | **.007** |  |  | -0.671 | 0.943 | -.091 | .479 |  |  | | -0.390 | | 0.788 | | | | -.055 | | .622 | | |  | | | | |  | | | |  |  |
| Number of PTSD cases | -0.031 | 0.036 | -.120 | .386 |  |  | 0.029 | 0.033 | .130 | .379 |  |  | | 0.057 | | 0.028 | | | | .264 | | **.041** | | |  | | | | |  | | | |  |  |
| Therapy sessions per week | -0.005 | 0.062 | -.010 | .936 |  |  | -0.083 | 0.058 | -.181 | .153 |  |  | | -0.156 | | 0.048 | | | | -.352 | | **.002** | | |  | | | | |  | | | |  |  |
| AT (trauma therapy) | 0.645 | 1.175 | .074 | .585 |  |  | 1.520 | 1.087 | .202 | .166 |  |  | | 2.442 | | 0.909 | | | | 0.337 | | **.009** | | |  | | | | |  | | | |  |  |
| AT (schema therapy) | -2.886 | 1.570 | -.215 | .070 |  |  | -0.909 | 1.453 | -.078 | .533 |  |  | | -0.791 | | 1.214 | | | | -.071 | | .517 | | |  | | | | |  | | | |  |  |
| AT (hypnotherapy) | -3.197 | 1.740 | -.203 | .070 |  |  | -0.136 | 1.609 | -.010 | .933 |  |  | | -0.760 | | 1.345 | | | | -.058 | | .574 | | |  | | | | |  | | | |  |  |
| AT (systemic therapy) | -1.139 | 1.316 | -.104 | .390 |  |  | 0.433 | 1.217 | .046 | .723 |  |  | | 1.400 | | 1.018 | | | | .152 | | .173 | | |  | | | | |  | | | |  |  |
| AT (EMDR) | 1.317 | 1.242 | .150 | .293 |  |  | -1.577 | 1.149 | -.208 | .174 |  |  | | -1.253 | | 0.961 | | | | -.171 | | .196 | | |  | | | | |  | | | |  |  |
| AT (yes / no) | -0.684 | 1.228 | -.077 | .579 |  |  | -0.063 | 1.136 | -.008 | .956 |  |  | | -1.187 | | 0.950 | | | | -.159 | | .215 | | |  | | | | |  | | | |  |  |
| PB (psychology) | 1.422 | 1.428 | .158 | .323 |  |  | -0.087 | 1.321 | -.011 | .948 |  |  | | -0.539 | | 1.105 | | | | -.072 | | .627 | | |  | | | | |  | | | |  |  |
| PB (social work) | 0.496 | 1.343 | .050 | .713 |  |  | -0.290 | 1.242 | -.034 | .816 |  |  | | 0.529 | | 1.038 | | | | .064 | | .612 | | |  | | | | |  | | | |  |  |
| PB (teaching) | 0.634 | 1.800 | .045 | .726 |  |  | -0.589 | 1.665 | -.048 | .724 |  |  | | 0.416 | | 1.392 | | | | .035 | | .766 | | |  | | | | |  | | | |  |  |
| PB (pedagogy) | 0.672 | 1.357 | .080 | .622 |  |  | 0.142 | 1.255 | .019 | .910 |  |  | | 0.651 | | 1.049 | | | | .092 | | .537 | | |  | | | | |  | | | |  |  |
| PB (social pedagogy) | 1.484 | 1.457 | .150 | .312 |  |  | 0.436 | 1.348 | .051 | .747 |  |  | | 1.350 | | 1.127 | | | | .163 | | .235 | | |  | | | | |  | | | |  |  |
| Therapeutic approach (depth psychology) | 0.002 | 3.004 | .000 | .999 |  |  | -0.961 | 2.779 | -.093 | .730 |  |  | | -0.520 | | 2.323 | | | | -.052 | | .823 | | |  | | | | |  | | | |  |  |
| Therapeutic approach (behavioral therapy) | 0.738 | 2.892 | .064 | .799 |  |  | -1.679 | 2.675 | -.167 | .532 |  |  | | 0.881 | | 2.237 | | | | .091 | | .695 | | |  | | | | |  | | | |  |  |
| Patients (children/youth) | -0.439 | 1.997 | -.028 | .827 |  |  | -0.871 | 1.847 | -.064 | .639 |  |  | | -1.646 | | 1.544 | | | | -.125 | | .290 | | |  | | | | |  | | | |  |  |
| Patients (adults) | -2.269 | 2.571 | -.123 | .380 |  |  | 1.429 | 2.378 | .090 | .550 |  |  | | 0.113 | | 1.988 | | | | .007 | | .955 | | |  | | | | |  | | | |  |  |
| *Note*. *b* = regression coefficient, *SE B* = standard error, *β* = standardized coefficient beta, *p* = significance, *R²* = correlation coefficient, *Adj. R²* = adjusted correlation coefficient. CS = compassion satisfaction, BO = Burnout, STS = Secondary traumatic stress, AT = Additional Training, PB = Professional Background, bold = significant values | | | | | | | | | | | | | | | | | | | | | | | | | | | | | | | | | | |  |
